# Bioactive Compounds and Sensory Analysis of Freeze-Dried Prickly Pear Fruits from An Inter-Andean Valley in Peru

**DOI:** 10.3390/molecules28093862

**Published:** 2023-05-03

**Authors:** David Choque-Quispe, Carlos A. Ligarda-Samanez, Edith R. Huamán-Rosales, John Peter Aguirre Landa, Henrry W. Agreda Cerna, Miluska M. Zamalloa-Puma, Genaro Julio Álvarez-López, Gloria I. Barboza-Palomino, Humberto Alzamora-Flores, Wilfredo Gamarra-Villanueva

**Affiliations:** 1 Water and Food Treatment Materials Research Laboratory, Universidad Nacional José María Arguedas, Andahuaylas 03701, Peru; 2 Agroindustrial Engineering Department, Universidad Nacional José María Arguedas, Andahuaylas 03701, Peru; caligarda@unajma.edu.pe (C.A.L.-S.); edithrosaluzhuamanrosales@gmail.com (E.R.H.-R.); 3 Research Group in the Development of Advanced Materials for Water and Food Treatment, Universidad Nacional José María Arguedas, Andahuaylas 03701, Peru; 4 Nutraceuticals and Biopolymers Research Group, Universidad Nacional José María Arguedas, Andahuaylas 03701, Peru; 5 Food Nanotechnology Research Laboratory, Universidad Nacional José María Arguedas, Andahuaylas 03701, Peru; 6 Business Administration Department, Universidad Nacional José María Arguedas, Andahuaylas 03701, Peru; jpaguirre@unajma.edu.pe (J.P.A.L.); hagreda@unajma.edu.pe (H.W.A.C.); 7 Physics Department, Universidad Nacional de San Antonio Abad del Cusco, Cusco 08000, Peru; miluska.zamalloa@unsaac.edu.pe; 8 Law and Humanities Faculty, Universidad Continental, Cusco 08000, Peru; galvarez@continental.edu.pe; 9 Chemical Engineering Department, Universidad Nacional de San Cristobal de Huamanga, Ayacucho 05000, Peru; gloria.barboza@unsch.edu.pe; 10 Education Department, Universidad Nacional de San Antonio Abad del Cusco, Cusco 08000, Peru; humberto.alzamora@unsaac.edu.pe; 11 Universidad Andina del Cusco, Cusco 08000, Peru; wgamarra@uandina.edu.pe

**Keywords:** freeze-drying, prickly pear ecotype, total polyphenols, antioxidant activity, vitamin C, color index, trace elements, sensory analysis

## Abstract

Prickly pear fruits are seasonal and have shades ranging from pale green to deep purple. Their pigments are associated with bioactive compounds, being sensitive to thermal transformation processes for their conservation. The objective of this research was to evaluate the bioactive compounds and the sensory analysis of freeze-dried prickly pear fruits from an inter-Andean valley in Peru. The prickly pear fruits of the morada, anaranjada, and blanca ecotypes came from an inter-Andean valley in Peru at 2972 m altitude. The sliced fruits were freeze-dried at −40 °C and 0.25 mTorr, and the total polyphenol content (TPC), vitamin C, and antioxidant activity (AA) were determined, as well as the color L* a* b*, color index (CI*), FTIR spectra, and mineral content. In the same way, sensory analysis of preferences with nine scales was applied. It was observed that in the freeze-dried fruits, TPC, AA, and vitamin C increased significantly (*p*-value < 0.05), and their corresponding functional groups increased in intensity in their corresponding FTIR spectra; furthermore, trace elements such as Cu, Fe, Se, Zn, Si, and Mn were identified. On the other hand, freeze-drying provided deeper colors to the fruits, which most panelists said they “very much liked” during the sensory analysis, although the texture was not very well accepted, with most panelists reporting being “indifferent” towards it. The freeze-drying technique allows the bioactive and sensory attributes of prickly pear fruits from inter-Andean valleys to be preserved, making it a potential fruit for export and conservation due to its seasonality.

## 1. Introduction

The *Opuntia ficus* indica is one of the most important cacti. They develop mainly in soil lacking nutrients and can tolerate low water demand; their production frequently extends to Central America, South America, South Africa, and part of the Mediterranean [1,2]. Its fruits are consumed directly or in juices, jams, and ice cream, and they can also be fermented, extruded, and transformed into flour [3,4,5].

The constituents of prickly pear (*Opuntia ficus* indica) fruits are subject to climatic conditions and soil composition. Numerous products that adapt to high altitude floors have higher levels of bioactive components. However, the size and quantity of production are minor [6,7,8,9,10].

Prickly pear fruits have a coloration ranging from intense purple to pale green. These shades are due to pigments derived mainly from nutraceutical and functional compounds, among which polyphenols, carotenoids, betalains, and vitamins stand out, acting as antioxidants when consumed [11,12,13,14]. In this way, they are present in fruits with antioxidant attributes, beneficial for health because they essentially inhibit the oxidative effects of reactive oxygen molecules [11,15,16]. These compounds prevent damage at the cellular level that can lead to diseases such as diabetes, cancer, cardiovascular problems, premature aging, and neurodegenerative disorders, which are due to oxidative stress caused by the imbalance of reactive oxygen species. Likewise, they have anti-inflammatory, anti-atherosclerotic, antiulcerogenic, hypoglycemic, immunomodulatory, and hepatoprotective properties [17,18,19].

It has been observed that the antioxidant activity of this fruit far exceeds that of some traditional fruits [20], which is why its consumption is becoming common, especially in European countries [13]. However, as it is a highly perishable product, its transportation and storage are crucial. These fruits are seasonal, and their availability in the market extends for very short periods.

On the other hand, these fruits are rich in polysaccharides, including pectins (high acetyl and low methoxyl content), arabinogalactans, rhamnogalactans, arabinoxylans, and rhamnogalaturans [21], being one of the few fruits with the presence of isorhamnetin glycosides, especially isorhamnetin-3-o-rutinoside and isorhamnetin triglucosides [6,22].

Different methods have been applied for the conservation of prickly pear fruits, mainly thermal; however, these compromise their beneficial properties, generally polyphenols and vitamins, due to contact with surrounding oxygen during some drying processes [23,24,25]. Likewise, they considerably influence sensory qualities, especially flavor and color. For this reason, using conservative methods that reduce the water level of these fruits is desirable, and freeze-drying offers advantages in terms of conserving the active constituents of a high-humidity fruit [26,27,28].

Therefore, the research objective was to study some bioactive compounds of prickly pear fruit dehydrated by freeze-drying from an inter-Andean valley, determining the antioxidant capacity and content of total phenols, vitamin C, and trace elements, as well as performing infrared analysis and sensory analysis of the freeze-dried fruits.

## 2. Results and Discussions

### 2.1. Determination of Physicochemical Parameters

**Figure 1:**
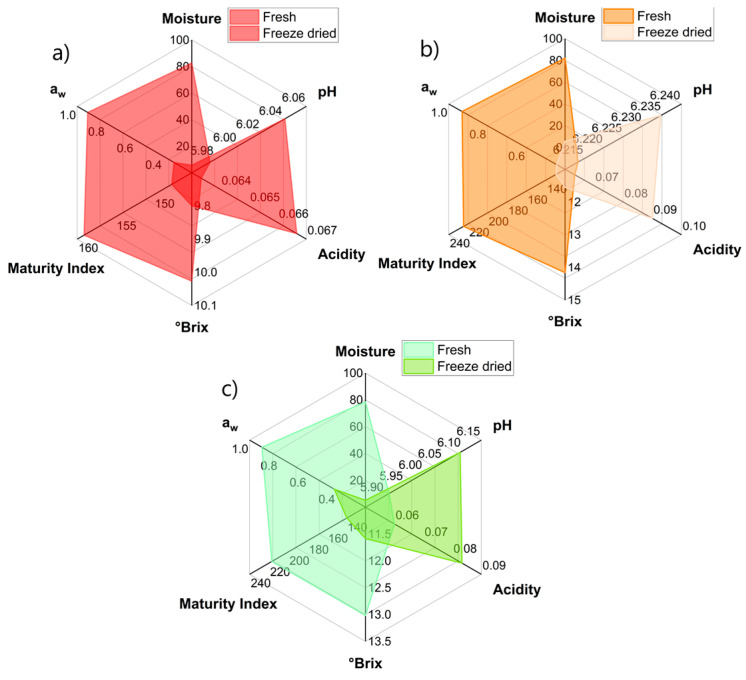
Variations in the physicochemical characteristics of fresh and freeze-dried prickly pear fruit: (**a**) morada, (**b**) anaranjada, (**c**) blanca.

Fresh prickly pear fruits have high moisture content [29], between 78.47% and 82.72% among the ecotypes (*p*-value < 0.05) (Table 1); this decreases considerably to values between 5.17% and 5.74% for freeze-dried fruits (*p*-value < 0.05). Regarding the pH, it was observed that it ranges from 5.91 to 6.21 for fresh fruits (*p*-value < 0.05) and increases slightly after freeze-drying (Figure 1).

On the other hand, the acidity was found to be around 0.06% in the fresh fruits, and it increased slightly after freeze-drying to 0.07–0.08% (*p*-value < 0.05); this increase is due to the arrest of the metabolic rate due to the freezing of the fruits prior to vacuum-drying and the elimination of water during drying [30,31].

As for soluble solids, these decreased slightly for the freeze-dried fruit (Figure 1); this is due to the enzymatic activity being stopped by the action of freezing and vacuum-drying, mainly those that hydrolyze complex sugars into mono and disaccharides [32]; this is reflected in the maturity index that decreases significantly (*p*-value < 0.05).

Regarding the water activity (*a_w_*), as expected, the fresh fruits presented a high value, making them susceptible to being attacked by microorganisms; however, these levels decrease considerably after freeze-drying, with values below 0.443 (Table 1), which would guarantee the preservation of the freeze-dried fruit [9].

### 2.2. Color

Lightness L* is associated with the brightness of a color [33,34] because water acts as a brightening agent in fresh fruits [35,36]. The morada ecotype, L*, decreased considerably for the freeze-dried samples from 42.17 to 28.77. This also happened for the anaranjada and blanca ecotypes, presenting more opaque colors (Table 2).

Regarding chroma a*, which would be attributed to phenols and polyphenols [37], which refers to colors in the range from green to red, it was found that they are in the positive quadrant for the morada and anaranjada ecotypes, with a greater red hue for the morada type (Table 2), although it decreased considerably after freeze-drying, whereas in the anaranjada type, it increased considerably.

For the blanca ecotype, a* was located in the negative quadrant, with a tendency towards green, being deeper after freeze-drying. Chroma b*, which is mainly attributed to the presence of carotenoids and chlorophylls [36,38], was found in the positive quadrant for all three ecotypes, with a considerable decrease for morada and anaranjada (*p*-value < 0.05), although there was a slight increase for the blanca ecotype.

**Figure 2:**
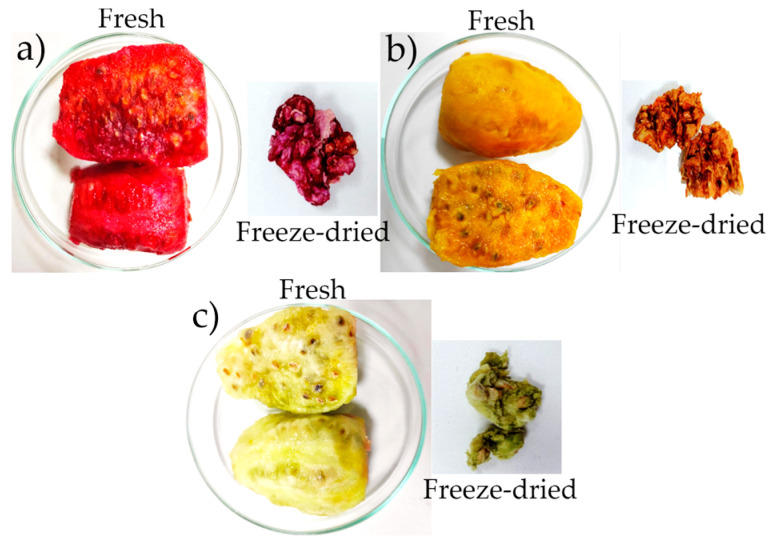
Fresh and freeze-dried fruits: (**a**) morada, (**b**) anaranjada, (**c**) blanca.

In general, the observed color tones of freeze-dried fruits were darker or deeper shades (Figure 2), as evidenced by calculating the color index [26]. In this sense, the freeze-drying technique allows for better preservation of the chromophoric components responsible for the color, such as phenols, vitamins, and betalains [11,12,13,39,40], because it avoids the combined effects of high temperature and oxygen during drying, maintaining the color sensory qualities of these fruits [41,42,43,44,45]. This behavior has been reported for high-humidity fruits subjected to the freeze-drying process [46,47,48,49,50].

### 2.3. Bioactive Compounds

An indicator of the presence of high total polyphenol content (TPC) is the intense coloration that the fruits present. It was observed that the fresh fruits of morada and anaranjada prickly pear had higher TPC, while the blanca prickly pear had 1026.74 mg EAG/100g d.b. (Table 3). After freeze-drying, a significant increase in TPC was observed (*p*-value < 0.05), being higher for the anaranjada prickly pear (1323.67 mg EAG/100g d.b.). The results suggest that these fruits are good sources of polyphenolic compounds [15]. This increase is due to the release of phenolic compounds trapped in the cell wall, as observed in blueberries and tomatoes. However, it could also decrease in other fruits due to the release of oxidative and hydrolytic enzymes that degrade phenolic compounds [48,51,52].

The capacity of prickly pear fruits to inhibit or eliminate free radicals was evaluated through the antioxidant capacity, which measures the capacity to donate hydrogen ions by DPPH [11,15,53]. It was observed that the anaranjada prickly pear had a higher value (3.25 µmol TE/100 g d.b.) and that it decreased considerably to 1.62 µmol TE/100 g d.b. (*p*-value < 0.05). In contrast, the morada prickly pear did not present a considerable decrease. Regarding the blanca prickly pear, it presented a lower value that was similar to that of the freeze-dried fruit (*p*-value > 0.05) (Table 3), and this is associated with the little pigmentation it presents.

Regarding vitamin C, it was observed that the fresh blanca prickly pear had higher content (50.01 mg/100 g d.b.). After submitting the fruits to freeze-drying, vitamin C content increased considerably (*p*-value < 0.05) for the three ecotypes; this is due to the low drying temperature since vitamin C is highly thermolabile, and the freeze-drying process is the most convenient for conserving this bioactive compound. It has been observed that the freeze-drying process minimizes the deterioration of this water-soluble vitamin in many fruits [48,54,55].

The presence of different bioactive compounds in foods and the nature of their phenolic groups is closely related to antioxidant activity. However, these may be affected by the measurement technique used [56] or by the conditions to which they are subjected during the transformation of food [57]. Therefore, if this activity is to be preserved, it is advisable to avoid the use of oxidizing substances or the uncontrolled use of temperature during the transformation of food. In this sense, freeze-drying is a means of food dehydration that guarantees the conservation of thermolabile and highly oxidizing bioactive compounds [27,28,37,39,42,44].

### 2.4. FTIR Analysis

**Figure 3:**
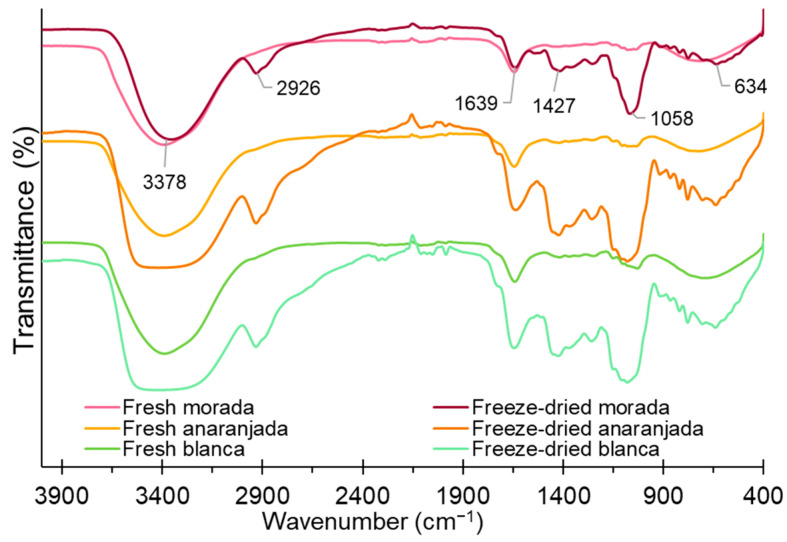
FTIR spectra for fresh and freeze-dried prickly pear fruits.

FTIR analysis allows the identification of functional groups of constituents in foods and is a quick measurement tool to identify certain compounds. Figure 3 shows a peak around 3378 cm^−1^, which corresponds to the strong stretching of the -OH and -NH groups and would be attributed to the presence of water, phenolic compounds, carbohydrates, vitamin C, and polypeptides [58,59], whose peaks present greater intensity in freeze-dried fruits; this is evidenced by the increase in bioactive compounds (TPC, AA, AA) shown in Table 3. Freeze-dried prickly pear fruits present a peak around 2926 cm^−1^, and this is due to the asymmetric stretching of the CH and NH_3_ groups due to the presence of amino acids and carboxylic acids. However, fresh fruits do not present such a peak given their high water content, which would considerably reduce their concentrations [41,60,61].

The peak around 1639 cm^−1^ presented greater intensity for freeze-dried fruits, which indicates the presence of -COO and -CO groups of phenolic compounds, flavonoids, and fats [62]. The peaks around 1058 to 1427 cm^−1^ indicate the presence of ether, esters, alcohols, and carboxylic acids [63] and present greater intensity for freeze-dried fruits. The peak at 634 cm^−1^ can be attributed to the -CH groups of the aromatic ring of polyphenols and polysaccharides [60]. It was observed that freeze-dried fruits improve and preserve the content of bioactive compounds; similar results were reported by Gouws et al. [27] and Henry et al. [41].

### 2.5. Mineral Content

Freeze-dried fruits are a significant source of K, Ca, and Mg, presenting values of 409.04–562.04, 95.13–119.13, and 34.13–37.06 mg/100 g, respectively, although Na was found in amounts between 0.53 and 2.74 mg/100 g (*p*-value < 0.05) (Table 4). Trace elements that are a vital source for various metabolic processes have been reported in freeze-dried fruits; Cu was found in amounts between 0.08 and 0.10 mg/100 g (*p*-value > 0.05). This is an element that plays an essential role in hemoglobin synthesis [64]; similar values were found by Bakar et al. [23] and Kivrak et al. [65]. Regarding Fe, which is an oxygen carrier for protein synthesis (hemoglobin and myoglobin) [66], it was found in amounts between 3.00 and 4.39 mg/100 g (*p*-value < 0.05), these values being characteristic of this fruit [7,29,65,67]. Se, a metalloid, was found in amounts between 3.83 and 5.10 mg/100 g; this element is essential in oxidative stress and its deficiency can cause heart disease, hypothyroidism, and problems in the immune system [68,69].

Zn is a cofactor of enzymes, and it plays an essential role in the health of skin, bones, teeth, hair, muscles, and brain function; it is also related to the immune system [70]. Freeze-dried fruits presented 4.48 to 4.92 mg/100 g of Zn; these values are characteristic of these fruits [23,65,71]. Mn presented values between 2.06 and 3.49 mg/100 g (*p*-value < 0.05); this trace element allows the proper functioning of the antioxidant system against free radicals, in addition to being a cofactor of enzymes such as pyruvate carboxylase and superoxide dismutase [72]. Likewise, Si was found in amounts between 2.94 and 3.47 mg/100 g.

Regarding Pb, it was observed that these amounts are below the limits established by the European Reg. 1881/2006 [73] for fresh fruits (1 mg/100 g). While the levels of Al and Ba are relatively low in the anaranjada ecotype, Pb was not detected at all. The fruits studied are an important source of trace elements such as Cu, Fe, Se, Zn, Si, and Mn. However, the mineral content generally depends on the growing conditions, soil type, ecotype, and water sources for their livelihood [7,67,71,74].

### 2.6. Sensory Analysis

During the sensory analysis, most panelists responded with “Like very much” in reference to the color and flavor attributes of the three ecotypes (Table 5); this is typical for freeze-dried products because the technique allows maintaining the qualities of the fruits and conserving the bioactive compounds [37,38]. Regarding the smell, the panelists generally responded “I like it slightly” or moderately. It is known that dehydrated fruits lose their smell considerably because the surface is more compact, avoiding the diffusion of aromas and odors into the environment. Regarding the texture, most panelists were “indifferent”; this was because the freeze-dried fruit introduced a sticky texture to the palate during chewing, attributed to the concentration of sugars such as fructose that do not crystallize. In this sense, freeze-drying is a widely used method to produce foods with high sensory quality, minimizing changes with respect to fresh fruit, even after rehydration [47].

### 2.7. PCA Study

**Figure 4:**
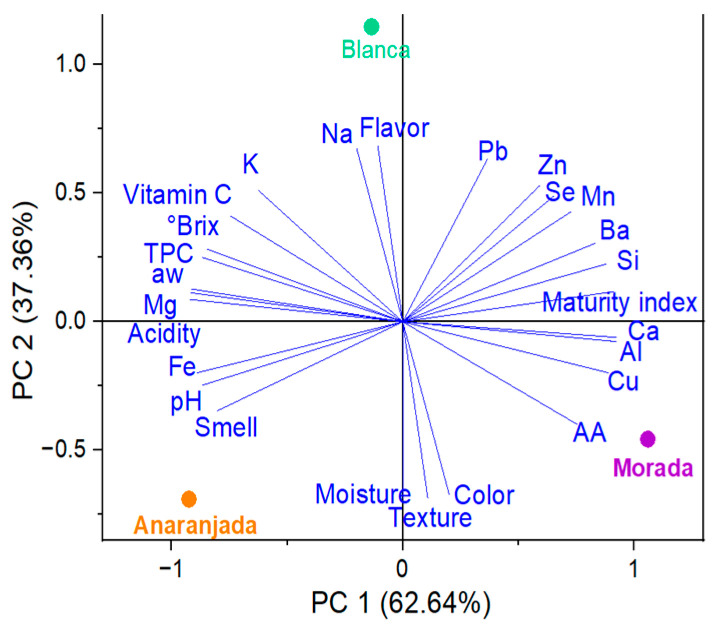
PCA for freeze-dried fruits.

The PCA results showed scattered trends of the parameters for the freeze-dried fruits studied. The blanca ecotype had higher Na, Zn, and Se content and better flavor (Figure 4). Regarding the morada ecotype, it stood out in Ca, Cu, Al, Si, and Ba content, with better color and texture attributes. The anaranjada fruit presented higher K, Mg, Fe, TPC, and vitamin C content, with high water activity, more soluble solids, and better pH, acidity, and odor.

## 3. Materials and Methods

### 3.1. Raw Materials

Three ecotypes of prickly pear fruits (*Opuntia ficus* indica) called morada, anaranjada, and blanca were collected from the Huancas Populated Center (13°30′29″ S, 73°22′57″ W, and 2972 m altitude) from the district of Andarapa, Andahuyalas, Peru, in the period October–December 2021.

### 3.2. Freeze-Drying

The shelled fruits were cut crosswise with an average thickness of 1 cm. On a stainless-steel plate, they were frozen at −20 °C for 24 h, then taken to an ilShin BioBase freeze-dryer, model TDF5503 (Rijssen, The Netherlands), set at −40 °C with 0.25 mTorr vacuum for 24 h. The samples were removed and placed in a desiccator for further analysis.

### 3.3. Determination of Physicochemical Parameters

Moisture, pH, soluble solids, and acidity on a dry basis (as a percentage of citric acid) were determined, according to Methods 934.06, 981.12, 932.12, and 942.15, respectively, proposed by the Association of Official Analytical Chemists [75].

The maturity index was determined as the ratio of soluble solids and acidity.

### 3.4. Water Activity (a_w_)

Samples were taken to a previously calibrated water activity (*a*_w_) determiner, Rotronic brand, model HygroPalm23-AW (Bassersdorf, Switzerland).

### 3.5. Fruit Color

The color was determined in the CIE L* a* b* space, with the following criteria: L* luminosity (0 = black and 100 = white), a* and b* chroma (+*a* = red, −*a* = green, +*b* = yellow, and −*b* = blue) [76]. The samples were placed on a glass plate and taken to a Konica Minolta colorimeter, model CR-5 (Tokyo, Japan). The readings were performed in the reflectance module.

In addition, the color index (IC*) was determined (Equation (1)), which allows color to be expressed in a single numerical datum [77], and is interpreted as follows:

- If IC* −40 to −20, colors range from blue-violet to deep green.
- If IC* −20 to −2, colors range from deep green to yellowish-green.
- If IC* −2 to +2, colors are greenish-yellow.
- If IC* +2 to +20, colors range from pale yellow to deep orange.
- If IC* +20 to +40, colors range from deep orange to deep red.

(1) $${IC}^{*}=\frac{a^{*}\times 1000}{L^{*}\times b^{*}}$$

### 3.6. Total Polyphenol Content (TPC)

Total polyphenol content (TPC) was determined using the Folin–Ciocalteu reagent at 0.25 N (Merck, Darmstadt, Germany) and 20% Na2CO3 (Spectrum, NB, Canada). A calibration curve was prepared from 5 to 35 mg/L gallic acid (Merck, Darmstadt, Germany), with R^2^ of 0.98.

In total, 0.9 mL of methanolic extract of the fruit was taken and mixed with 0.15 mL of Na_2_CO_3_, 0.30 mL of Folin–Ciocalteu reagent, and 0.90 mL of ultrapure water. The mixture was kept for 90 min in the dark and then taken to a UV spectrophotometer, Thermo Fisher brand, Genesys 150 model (Waltham, MA, USA), and absorbance readings were made at 750 nm. The results were expressed as mg/gallic acid equivalent (GAE) per 100 g of sample on a dry basis [78].

### 3.7. Antioxidant Capacity

The free radical method using DPPH reagent (Himedia, Mumbai, India) was used to determine antioxidant capacity. A calibration curve (R^2^ 0.98) was prepared with Trolox reagent (Sigma-Aldrich, MI, USA). In total, 0.15 mL of the extract (hydrophilic phase) was taken, and 2.85 mL of diluted DPPH was added. The mixture was stored in the dark at room temperature until measured.

The mixture was then taken to a Thermo Fisher brand UV spectrophotometer, Genesys 150 model (Waltham, MA, USA), at 515 nm. The results were expressed as mg Trolox equivalent (TE) per 100 g of sample on a dry basis (d.b.) [78].

### 3.8. Vitamin C

Vitamin C content was determined through AOAC Method 967.21 [75]. First, 0.5 g of the sample was weighed and macerated with 5 mL of 1% metaphosphoric acid (Merck, Darmstadt, Germany) for 45 min; then, it was filtered and calibrated to 5 mL. Next, 1 mL of the extract was taken and diluted in 9 mL of 2,6-dichloroindophenol (12 mg/L) (Merck, Darmstadt, Germany). Absorbance was read at 515 nm with a Thermo Fisher brand UV spectrophotometer, model Genesys 150 (Waltham, MA, USA), and deionized water was used as a blank. The results were expressed in mg of ascorbic acid (AA) per 100 g on a dry basis.

### 3.9. IR Analysis

Fruit tablets were prepared at 0.1% KBr (IR Grade, Darmstadt, Germany). They were brought to the transmission module of the FTIR spectrometer (Fourier transform infrared spectroscopy), Thermo Fisher (Waltham, MA, USA), Nicolet IS50 model, in a range of 4000 to 400 cm^−1^ with a resolution of 4 cm^−1^ and 32 scans.

### 3.10. Mineral Determination

In total, 500 mg of the samples was digested at 180 °C for 20 min in an acid medium (12 mL of 65% nitric acid/3 mL of 37.7% hydrochloric acid) gauged with ultrapure water to 50 mL in a microwave digester, SCP Science brand, MiniWave model (Quebec, Canada). The digested samples were filtered at 0.45 µm and an aliquot was taken for metal quantification in an inductively coupled plasma optical emission spectrometer, Shimadzu brand, model ICP-OES 9820 (Kyoto, Japan). Calibration curves were previously prepared for the metals under study, with a regression coefficient R^2^ > 0.995. Aliquots were analyzed in axial mode with 10 L/min argon gas flow and 30 s plasma exposure, with a 30 s rinse at 60 rpm between samples [79].

### 3.11. Sensory Analysis

A preference test for color, taste, smell, and texture was carried out using a hedonic scale that allowed measuring whether the freeze-dried fruit was liked or disliked: (9) Extremely like, (8) Like very much, (7) Moderately like, (6) Like slightly, (5) Neither like nor dislike, (4) Dislike slightly, (3) Dislike moderately, (2) Dislike very much, (1) Dislike extremely. Fifty semi-trained judges of both sexes aged between 18 and 50 years participated in the sensory analysis.

### 3.12. Statistical Analysis

The significant difference in the data was analyzed through an ANOVA, and a Tukey test (HSD) was also applied. Regarding the sensory analysis, a Kruskal–Wallis test was applied since the data presented a non-normal distribution (*p*-value < 0.05) evaluated through the Shapiro–Wilk test. The statistical inference of the data was analyzed at 5% significance. In the same way, a principal component analysis (PCA) was carried out in order to know the trend of each freeze-dried fruit. The Origin Pro 2023 program (OriginLab Corporation, Northampton, MA, USA) was used for the graphical representation and statistical tests, and Excel spreadsheets.

## 4. Conclusions

The fresh prickly pear fruits of the morada, anaranjada, and blanca ecotypes grown in the inter-Andean valleys have good bioactive compound content, particularly polyphenols and vitamin C. When subjected to freeze-drying, the three ecotypes preserve and improve their bioactive qualities, slightly increasing the levels of TPC, AA, and vitamin C, which was confirmed by the FTIR analysis, where the spectra for these compounds presented greater intensity. The mineral analysis reported high levels for K > Ca > Mg > Se > Zn > Fe, with a good content of trace elements. In the same way, the freeze-drying of fruits maintained their color and even provided deeper colors that were accepted by the sensory analysis panelists, who responded with “Like very much” in reference to the color, although the texture was not widely accepted, with panelists reporting they were “indifferent” towards it. The freeze-drying technique allows the bioactive and sensory attributes of prickly pear fruits from the inter-Andean valleys to be preserved considerably, making it a potential fruit for export and conservation, since it is seasonal.

## Figures and Tables

**Table 1:** Physicochemical characteristics of fresh and freeze-dried prickly pear fruit.

	Morada				Anaranjada				Blanca			
	$\overset{-}{x}$	±SD	CV	*	$\overset{-}{x}$	±SD	CV	*	$\overset{-}{x}$	±SD	CV	*
Fresh												
Moisture	82.72	0.60	0.72	a	82.06	0.75	0.91	a	78.47	0.62	0.79	b
pH	5.98	0.60	10.01	a	6.21	0.75	12.05	b	5.91	0.62	10.48	c
Acidity (%)	0.06	0.01	9.12	a	0.06	0.01	9.12	a	0.06	0.00	0.00	a
Soluble solids (°Brix)	10.03	0.05	0.46	a	14.16	0.17	1.23	b	13.02	0.03	0.22	c
Maturity index	159.11	13.09	8.22	a	224.60	16.85	7.50	b	216.94	0.48	0.22	b
aw	0.927	0.002	0.165	a	0.931	0.001	0.107	b	0.915	0.001	0.109	c
Freeze-dried												
Moisture	5.67	0.02	0.35	a	5.74	0.05	0.89	a	5.17	0.03	0.55	b
pH	6.04	0.02	0.33	a	6.23	0.05	0.82	b	6.10	0.03	0.46	c
Acidity (%)	0.07	0.01	8.66	a	0.09	0.01	11.11	b	0.08	0.01	6.93	a, b
Soluble solids (°Brix)	9.80	0.17	1.70	a	11.55	0.16	1.39	b	11.58	0.24	2.04	b
Maturity index	147.63	10.87	7.36	a	129.28	12.79	9.89	a	139.31	6.64	4.77	a
aw	0.324	0.001	0.309	a	0.443	0.000	0.000	b	0.416	0.002	0.556	c

$\overset{-}{x}$ is the arithmetic mean; SD is the standard deviation; CV is the variability coefficient (%). * Different letters in the rows indicate significant difference, evaluated through ANOVA and Tukey’s test at 5% significance, for *n* = 3.

**Table 2:** Colors of fresh and freeze-dried fruits.

Ecotype		L*				a*				b*				IC*				
		$\overset{-}{x}$	±SD	CV	**	$\overset{-}{x}$	±SD	CV	**	$\overset{-}{x}$	±SD	CV	**	$\overset{-}{x}$	±SD	CV	**	
Morada	Fresh	42.17	0.40	0.96	<0.05	67.67	0.57	0.84	<0.05	20.73	0.40	1.95	<0.05	77.42	1.50	1.94	<0.05	
	Freeze dried	28.77	0.31	1.06		51.47	0.38	0.74		13.87	0.21	1.50		129.04	1.24	0.96		
Anaranjada	Fresh	75.87	0.46	0.60	<0.05	19.93	0.20	1.00	<0.05	78.07	0.25	0.32	<0.05	7.84	0.14	1.74	<0.05	
	Freeze dried	60.60	0.59	0.97		32.10	0.32	1.00		67.53	0.25	0.37		3.37	0.05	1.41		
Blanca	Fresh	93.57	0.32	0.34	<0.05	-29.27	0.50	1.72	<0.05	25.10	0.20	0.80	<0.05	-12.46	0.16	1.27	<0.05	
	Freeze dried	86.73	0.42	0.48		-38.07	0.45	1.18		27.33	0.25	0.92		-16.06	0.25	1.58		

$\overset{-}{x}$ is the arithmetic mean; SD is the standard deviation; CV is the variability coefficient (%). ** Evaluated through ANOVA at 5% significance for *n* = 5.

**Table 3:** Total polyphenols, antioxidant activity, and vitamin C of fresh and freeze-dried fruits.

Ecotype		TPC (mg EAG/100 g d.b.)		AA (µmol TE/100 g d.b.)						Vitamin C (mg /100 g d.b.)			
		$\overset{-}{x}$	±SD	CV	*	$\overset{-}{x}$	±SD	CV	*	$\overset{-}{x}$	±SD	CV	*
Morada	Fresh	1221.33	1.26	0.10	>0.05	3.04	0.01	0.20	<0.05	27.23	0.64	2.36	<0.05
	Freeze-dried	1247.91	17.58	1.41		3.41	0.08	2.30		30.14	0.59	1.97	
Anaranjada	Fresh	1163.29	0.61	0.05	<0.05	3.25	0.10	3.12	<0.05	39.29	0.67	1.72	<0.05
	Freeze-dried	1323.67	13.19	1.00		1.62	0.01	0.48		47.01	0.45	0.96	
Blanca	Fresh	1026.74	4.38	0.43	<0.05	1.12	0.02	2.07	>0.05	50.01	0.82	1.64	<0.05
	Freeze-dried	1320.58	17.30	1.31		1.11	0.07	6.11		52.33	1.00	1.90	

$\overset{-}{x}$ is the arithmetic mean; SD is the standard deviation; CV is the variability coefficient (%). * Evaluated through ANOVA at 5% significance for *n* = 3.

**Table 4:** Minerals in freeze-dried prickly pear fruits.

Mineral (mg/100 g d.b.)	Wavelength, nm	Morada				Anaranjada				Blanca			
		$\overset{-}{x}$	±SD	CV	*	$\overset{-}{x}$	±SD	CV	*	$\overset{-}{x}$	±SD	CV	*
Al	396.15	0.57	0.05	9.12	a	0.19	0.05	24.12	b	0.30	0.03	8.30	c
Ba	455.40	0.44	0.01	1.14	a	ND				0.36	0.01	1.40	b
Ca	183.80	119.13	0.06	0.05	a	95.13	1.00	1.05	b	102.80	0.61	0.59	c
Cu	324.75	0.10	0.02	20.15	a	0.08	0.02	27.15	a	0.08	0.01	12.50	a
Fe	259.94	3.00	0.01	0.19	a	4.39	0.02	0.47	b	3.48	0.04	1.16	c
K	766.49	409.04	4.58	1.12	a	498.37	2.35	0.47	b	562.04	2.09	0.37	c
Mg	285.21	34.13	0.26	0.76	a	37.06	0.20	0.55	b	36.33	0.20	0.54	c
Na	588.99	0.53	0.04	6.88	a	0.77	0.03	4.45	b	2.74	0.00	0.02	c
Pb	220.35	0.05	0.01	20.00	a	0.04	0.01	13.32	a	0.06	0.01	10.19	a
Se	196.09	4.90	0.26	5.40	a	3.83	0.21	5.43	b	5.10	0.17	3.40	a
Zn	202.55	4.81	0.01	0.12	a	4.48	0.02	0.34	b	4.92	0.01	0.20	c
Si	212.41	3.47	0.12	3.43	a	2.94	0.12	4.01	b	3.31	0.12	3.61	a
Mn	257.61	3.45	0.03	0.93	a	2.06	0.02	0.74	b	3.49	0.01	0.17	a

$\overset{-}{x}$ is the arithmetic mean; SD is the standard deviation; CV is the variability coefficient (%); ND is not detected. * Different letters in the rows indicate significant differences, evaluated through an ANOVA and Tukey’s test at 5% significance for *n* = 3.

**Table 5:** Score and criteria of sensory attributes of freeze-dried fruits.

Attribute	Morada			Anaranjada			Blanca		
Color	7.5	a	Like very much	7.4	a	Like very much	6.6	b	like moderately
Flavor	7.7	a	Like very much	7.7	a	Like very much	7.8	a	Like very much
Smell	6.1	a	Like slightly	6.9	b	like moderately	6.2	a	Like slightly
Texture	4.8	a	Indifferent	4.8	a	Indifferent	4.7	a	Indifferent

## Data Availability

The data presented in this study are available in this article.

## Sample Availability

Samples of the fruits under study; may be available from the authors.
